# Neuraxial anesthesia versus general anesthesia for cesarean delivery: Maternal and neonatal outcomes

**DOI:** 10.6026/973206300221131

**Published:** 2026-02-28

**Authors:** Pratibha Rathore, Bhawna Kansal, Romesh Sharma, Sona Joseph, Gourab Biswas, Ishika Chakarvarti

**Affiliations:** 1 Department of Obstetrics and Gynaecology, Sri Rawatpura Sarkar Institute of Medical Sciences And Research, Raipur Chhattisgarh, India; 2 Department of Obstetrics and Gynaecology, NCR IMS Nalpur Meerut, Uttar Pradesh, India; 3 Department of Anaesthesiology, Sri Rawatpura Sarkar Institute of Medical Sciences and Research, Raipur, Chhattisgarh, India; 4 Department of Obstetrics and Gynaecology, Christian Medical College Hospital, Vellore, Tamil Nadu, India; 5 Department of Orthopaedics, Max Superspeciality Hospital, Delhi NCR, India; 6 Department of Obstetrics and Gynaecology, Kasturba Medical College, Mangalore, Karnataka, India

**Keywords:** Cesarean delivery, neuraxial anesthesia, general anesthesia, maternal outcomes, neonatal outcomes, Apgar score, NICU admission, obstetric anesthesia

## Abstract

Cesarean delivery is one of the most frequently performed surgical procedures worldwide, and uncertainty persists regarding the optimal anesthetic technique to improve maternal and neonatal outcomes. This narrative review synthesizes current evidence comparing neuraxial and general anesthesia for cesarean delivery using data from major biomedical databases. Available evidence indicates that neuraxial anesthesia is associated with lower maternal morbidity, fewer anesthesia-related complications, and generally more favorable neonatal outcomes, including higher Apgar scores and reduced need for resuscitation or NICU admission. General anesthesia, although essential in specific emergency situations or when neuraxial techniques are contraindicated, is linked to increased maternal risks and certain neonatal respiratory complications. Overall, neuraxial anesthesia remains the preferred technique for most cesarean deliveries, while further prospective studies with standardized long-term outcomes are required to optimize anesthetic decision-making.

## Background:

The term "cesarean delivery" describes the birth of a fetus via uterine (hysterotomy) and abdominal wall (laparotomy) surgical incisions. When vaginal birth is not practical or safe, the procedure-which was first carried out as a last-ditch effort to preserve the fetus from a moribund mother-has developed into a common surgical intervention used to handle maternal or fetal problems. In order to maximize outcomes for both the mother and the fetus, cesarean birth has evolved over time from an emergency, doctor-driven intervention to a carefully thought-out process involving shared decision-making between the doctor and the patient [1]. The most common surgical operation in the world is caesarean section delivery (CD). Worldwide health systems have seen an increase in the number of cesarean deliveries since the procedure was first described around 400 years ago [2]. Internationally, there has been a noticeable and ongoing rise in the number of cesarean deliveries in recent decades, with significant regional variation [3]. Significant growth has been noted elsewhere, but the rate of increase has stayed comparatively low in many sub-Saharan African nations [4]. For instance, preventive therapeutic practices and medico-legal considerations contributed to the around 30% cesarean delivery rates in the United States by 2006 [5]. The prevalence of cesarean deliveries varies greatly throughout Europe, from 52.2% in Cyprus to 14.8% in Iceland, while in the UK, rates range from 24.6% in England to 29.9% in Northern Ireland [6]. In a similar vein, the number of caesarean deliveries in Australia increased from less than 20% in 1998 to about 30% by 2008 [7]. Additionally, rising tendencies have been seen across Asia, including Bangladesh, China, India and Nepal [7]. Despite the lack of reliable evidence for proportionate mother or newborn benefit, the sharp increase in cesarean delivery rates worldwide has become a major public health concern [8]. Despite the potential for life-saving measures, narrative evidence highlights that cesarean deliveries should only be performed when medically recommended because of the well-documented negative effects of procedure-related complications on maternal and neonatal morbidity [9, 10]. With reported maternal mortality rates of 35.9 deaths per 100,000 live deliveries compared to 9.2 deaths per 100,000 live births among women undergoing vaginal delivery, data compiled by the American College of Obstetricians and Gynecologists (ACOG) show that cesarean delivery is linked to a significantly higher risk of pregnancy-related morbidity and mortality [11]. Numerous negative health outcomes, such as childhood obesity, respiratory disorders, type 1 diabetes, acute lymphoblastic leukemia, impaired cognitive development, higher rates of autism and an increased risk of neurodevelopmental disorders, have been linked to infants born via cesarean delivery, according to some evidence [12, 13]. While neonatal sepsis, early neonatal death, stillbirth, perinatal asphyxia, low Apgar scores and prematurity are common neonatal complications, postpartum fever, surgical site infection, puerperal sepsis and maternal mortality are reported maternal outcomes linked to cesarean sections [14]. An estimated fourfold increase in the risk of maternal death is linked to cesarean birth, according to narrative evidence [15]. Moreover, needless caesarean deliveries have been associated with higher health care expenses, especially in low-income environments. The World Health Organization (WHO) advises that, at the population level, the rate of cesarean deliveries should not surpass 10% to 15% in response to these worries [16]. Globally, there are notable differences in the rates of cesarean sections between continents, nations, regions, cities and medical facilities. Numerous factors, including as socioeconomic position, poverty, discrepancies between rural and urban areas, the sophistication of healthcare systems, changes in maternal demographics and clinical characteristics and access to public and private healthcare facilities, have been implicated in these variances [11]. Therefore, it is of interest to describe caesarean section techniques.

## Caesarean section surgical techniques:

The term "caesarean section techniques" refers to any medically authorized treatment that involves cutting the skin, entering the abdomen, cutting the uterus, delivering the fetus, placenta and membranes and then closing and repairing the tissue layers. Nonetheless, there isn't a single best method for caesarean sections that everyone agrees with. Before conducting a cesarean section, a few common protocols should be followed regardless of the technique used. Informed verbal or written agreement that outlines the advantages, dangers and potential complications-including the possibility of a hysterectomy in extreme cases-must be obtained through proper patient preparation and counseling. In addition, a thorough medical and surgical history must be obtained, a physical examination must be performed, current medications and allergies must be reviewed, laboratory tests must be evaluated and in high-risk cases like major placenta praevia, placenta accreta or percreta, previous myomectomy or repeat cesarean delivery, a senior obstetrician and anesthetist must be available [18]. The clinical status of the patient determines whether regional or general anesthesia is used. In addition to lowering the risk of maternal and fetal problems, using suitable and efficient anesthetic during a cesarean section also lowers the risk of intraoperative mother awareness. Emergency situations and maternal refusal of regional anesthesia, such as coagulation or spinal abnormalities, are the most frequent reasons for general anesthesia. Placenta previa and other obstetric indications were regarded as absolute grounds for general anesthesia [19]. For the majority of patients having cesarean sections, neuraxial anesthesia is currently recommended as the reference procedure rather than general anesthesia [20].

## Materials and Methodology:

## Study design:

This study was conducted as a narrative review of the literature to synthesize and critically appraise existing evidence comparing neuraxial anesthesia (spinal, epidural, or combined spinal-epidural) with general anesthesia for cesarean delivery, with a focus on maternal and neonatal outcomes. A narrative approach was selected to allow contextual interpretation of heterogeneous study designs, clinical scenarios and patient populations, which are not always amenable to strict quantitative pooling.

## Literature search strategy:

A comprehensive literature search was performed across major biomedical databases, including PubMed/MEDLINE, Embase, the Cochrane Library, Web of Science and Scopus, covering publications from database inception until June 2025. The search strategy combined Medical Subject Headings (MeSH) and free-text terms related to anesthetic techniques and cesarean delivery.

Key search terms included:

[1] neuraxial anesthesia, spinal anesthesia, epidural anesthesia, combined spinal-epidural

[2] general anesthesia

[3] cesarean section or cesarean delivery

[4] maternal outcomes, neonatal outcomes, Apgar score, NICU admission, blood loss, hemodynamic stability

Boolean operators ("AND", "OR") were applied to refine the search.

## Eligibility criteria:

Studies were considered eligible for inclusion if they met the following criteria:

## Inclusion criteria:

[1] Randomized controlled trials, prospective or retrospective observational studies and comparative cohort studies

[2] Studies comparing neuraxial anesthesia with general anesthesia for cesarean delivery

[3] Reporting at least one relevant maternal outcome (e.g., blood loss, hemodynamic stability, postoperative recovery, ICU admission) and/or neonatal outcome (e.g., Apgar scores, resuscitation requirement, neonatal asphyxia, NICU admission)

## Exclusion criteria:

[1] Case reports, narrative commentaries, editorials, conference abstracts without full data

[2] Studies lacking a comparative anesthetic group

[3] Articles not reporting clinically relevant maternal or neonatal outcomes

[4] Non-English language publications where reliable translation was unavailable

## Study selection:

Titles and abstracts retrieved from the search were screened for relevance. Full texts of potentially eligible studies were reviewed to determine final inclusion. Given the narrative design, emphasis was placed on methodological relevance, clinical applicability and consistency of reported outcomes rather than strict quantitative homogeneity.

## Data extraction and synthesis:

From each included study, relevant data were systematically extracted, including the author and year of publication, study design and setting, sample size and key patient characteristics. Information on the type of anesthesia administered (general versus neuraxial techniques) and the indication for cesarean delivery (elective or emergency) was recorded. Reported maternal outcomes such as intraoperative blood loss, transfusion requirements, hemodynamic changes, postoperative recovery and intensive care unit admission were collected alongside neonatal outcomes, including Apgar scores, need for resuscitation, occurrence of neonatal asphyxia and neonatal intensive care unit admission. Data extraction was conducted in a structured and standardized manner to ensure consistency across studies. The extracted findings were then qualitatively synthesized and organized thematically, enabling comparison of outcome trends across studies while accounting for variations in clinical context, study design and patient risk profiles.

## Ethical considerations:

As this study was based exclusively on previously published data, no ethical approval or informed consent was required.

## Neuraxial anesthesia versus general anesthesia:

General anesthetic (GA) rates for both emergency and elective cesarean sections have occasionally surpassed standards suggested by professional organizations like the Royal College of Anaesthetists, according to a number of publications from obstetric anesthesia practice. Concerns were expressed about the quality of perioperative communication before an emergency cesarean delivery as well as the frequency of GA usage in one institutional experience that spanned the late 1990s and early 2000s. Significant gaps in cooperation and information sharing during the perioperative phase were found by a subsequent multidisciplinary evaluation, which may have influenced the choice of anesthetic. Targeted interventions that improved interprofessional collaboration and communication pathways were linked to a subsequent decrease in GA consumption, even if the available data was not enough to definitively assess the appropriateness of GA indications in individual cases [17,18]. In urgent and emergency cesarean procedures, where a number of unfavorable circumstances may affect infant status, Cocchi *et al.* assessed the impact of anesthetic type on both immediate and long-term neonatal outcomes. 395 women who had non-elective caesarean deliveries between 2021 and 2023 had their outcomes examined. The results showed that neonatal outcomes, such as lower Apgar scores at one minute, greater rates of NICU admission and increased likelihood of neonatal resuscitation, were considerably poorer when general anesthesia was used. These findings underline the significance of anesthesia selection and bolster the preference for neuraxial procedures by showing that general anesthesia in non-elective cesarean sections is linked to higher newborn morbidity that lasts past the early resuscitation period [20]. Using cesarean delivery cases performed in New York State hospitals between January 2006 and December 2013, Guglielminotti and Li evaluated the relationship between general anesthesia and unfavorable psychiatric outcomes in a retrospective cohort analysis. 34,356 (8.0%) of the 428,204 caesarean births were carried out under general anesthesia. 1158 women had severe PPD that required hospitalization; the majority of these instances were discovered during readmission, which occurred 164 days after discharge on average. While there was no discernible correlation with anxiety disorders or posttraumatic stress disorder, general anesthesia was linked to higher probabilities of PPD and suicidal thoughts or self-harm when compared to neuraxial anesthesia [21]. When general anesthesia is used during cesarean birth instead of neuraxial anesthesia, there is a continuously higher chance of negative consequences for the mother. General anesthesia use is associated with increased incidence of maternal death, cardiac arrest, anesthesia-related complications and surgical site infections, according to evidence compiled in the reviewed study [22]. Along with these grave side effects, general anesthesia has also been linked to a higher risk of venous thromboembolic events, such as pulmonary embolism and deep vein thrombosis. In addition, the article's population-based analysis showed that, when compared to neuraxial techniques, cesarean deliveries carried out under general anesthesia without a documented clinical indication were linked to significantly higher odds of anesthesia-related complications, severe anesthesia complications, surgical site infection and venous thromboembolism. The persistent correlation between general anesthetic and various types of maternal morbidity underscores its adverse risk profile, even if no statistically significant rise in the composite result of maternal mortality or cardiac arrest was noted in this cohort. Professional guidelines that discourage the routine use of general anesthesia for cesarean deliveries when neuraxial anesthesia is feasible were largely based on these unfavorable outcome data [23]. The chances of maternal adverse events are considerably higher when general anesthesia is used for cesarean delivery as opposed to neuraxial anesthesia. These include surgical site infections, anesthesia-related problems, cardiac arrest and death [22]. There is also evidence of an elevated risk of pulmonary embolism and deep vein thrombosis [23]. The American Society of Anesthesiologists (ASA) Practice Guidelines for Obstetric Anesthesia from 2007 and 2016 took these increased risks of maternal adverse events into account, which is why the statements "neuraxial techniques are preferred to general anesthesia for most cesarean deliveries" and "consider selecting neuraxial techniques in preference to general anesthesia for most cesarean deliveries" [24] were made. Unintentional intravascular injection rates of 1 in 5000 and high spinal rates of 1 in 16,000 were reported in a prospective multicenter study of 145550 epidurals performed in the UK between 1987 and 2003 [25]. High spinal in 23 cases (0.07%) and no occurrences of meningitis, neuraxial abscess or central nervous system hematoma were reported in a prospective multi- center research conducted in the USA of approximately 34600 women who underwent cesarean birth using neuraxial procedures between 1999 and 2002 [26]. According to a Cochrane analysis of 16 studies, "no significant difference was seen in terms of neonatal Apgar scores of 6 or less and of 4 or less at 1 and 5 minutes and need for neonatal resuscitation" between neuraxial blockade and general anesthesia in otherwise straightforward Cesarean deliveries [27]. In a research evaluating magnesium sulfate for preventing cerebral palsy in infants at risk of preterm birth, Robbins *et al.* (2021) compare the results of general versus neuraxial anesthesia in women undergoing cesarean delivery. The overall composite outcome of developmental delay did not significantly differ between the two anesthetic groups, according to comparative analysis. Similar results between groups were also found by subdomain analysis of developmental outcomes. However, babies subjected to general anesthesia had a greater chance of experiencing a severe motor delay. Furthermore, neonatal intensive care unit stays were greater for neonates in the general anesthesia group. Although certain negative motor outcomes and longer neonatal care were observed, the results indicate that general anesthesia for cesarean delivery was not linked to an increased risk of overall neurodevelopmental delay at two years. To learn more about the long-term neurodevelopmental effects of anesthetic exposure, more study is advised [28]. In order to assess maternal complications and neonatal outcomes related to general anesthesia for cesarean delivery, Bao *et al.* performed a retrospective analysis using data obtained from an Electronic Health Record System between January 2013 and December 2016. The rate of general anesthesia for caesarean deliveries rose dramatically during the research period. The percentage of repeat cesarean deliveries among women under general anesthesia also increased and one significant pregnancy-related complication in this group was the development of a morbidly attached placenta. The usage of laryngeal mask airways has significantly increased as airway management techniques have changed over time. Significant differences in neonatal outcomes between the groups receiving general and neuraxial anesthesia were revealed by comparative analysis. These differences included lower Apgar scores at one and five minutes, variations in birth weight and higher rates of neonatal intensive care unit admission among infants receiving general anesthesia. Overall, compared to neuraxial anesthesia, the growing use of general anesthetic was linked to worse newborn outcomes, higher rates of repeat cesarean deliveries and morbidly adherent placentas [29]. A retrospective cross-sectional single- center study by Thomas *et al.* examined 35,117 caesarean births from 2007 to 2018 in order to assess racial and ethnic disparities in the use of general anesthesia. We reviewed maternal demographics, obstetric features, anesthetic information and anesthesia and cesarean delivery indications. Overall, neuraxial anesthesia continued to be the most common technique, with general anesthesia being utilized in 3.3% of patients. Compared to Asian and non-Hispanic White patients, Black and Hispanic patients had higher rates of general anesthesia. There were no notable racial or ethnic variations between the subset of laboring patients who had neuraxial labor analgesia in situ and those who continued neuraxial anesthesia and those who converted to general anesthesia. All racial and ethnic groups had similar indications for anesthetic and cesarean delivery. While there were no racial differences in the use of neuraxial anesthesia when epidural analgesia was previously established, the results show persisting overall disparities in the use of general anesthesia [30]. The effects of general anesthesia and neuraxial anesthesia on mother and newborn outcomes after emergency cesarean sections carried out between January 2015 and July 2021 were assessed in a retrospective cohort research. Compared to newborns delivered under neuraxial anesthesia, those delivered under general anesthesia had lower Apgar scores at 1 and 5 minutes. Additionally, general anesthesia was linked to greater rates of infant respiratory distress syndrome, neonatal resuscitation and neonatal intensive care unit admission. While the period from the beginning of anesthesia to the incision was longer with neuraxial procedures, the decision-to-delivery and decision-to-incision intervals were similar for general anesthesia and epidural anesthesia. The length of a neonatal intensive care unit stay and other neonatal problems did not significantly differ between anesthetic modalities. Overall, in emergency cesarean deliveries, neuraxial anesthesia showed similar procedural timing to general anesthesia and was not linked to unfavorable outcomes for either the mother or the newborn, indicating that it could be a useful substitute for general anesthesia in specific clinical settings [31].

## Challenges:

Innovation, adaptation and certain adjustments to the role of surgeons, particularly in the procedure of caesarean sections, will be necessary in the future due to scientific and technological advancements, particularly in robotic surgery and minimal access surgery. At the moment, laparoscopy and robotic surgery are only used to treat cholecystectomy, ectopic scars after caesarean sections and scar dehiscence following cesarean sections. The Royal College of Surgeons has, however, recently raised the possibility of a robotic cesarean section. Comparing neuraxial with general anesthesia for cesarean delivery still presents a number of difficulties. Significant selection bias can be introduced by variations in patient comorbidities, surgical urgency and clinical indications, particularly in retrospective research. The choice of anesthesia and its results may be influenced by variations in practitioner experience and institutional procedures. Time restrictions frequently favor general anesthesia in emergency situations, which can complicate comparisons of maternal and newborn outcomes. Furthermore, direct comparison between studies is limited by the variety of outcome measures such perioperative parameters, neurodevelopmental assessment and Apgar scores. Long-term newborn and maternal consequences are especially difficult to grasp due to a lack of long-term follow-up data.

## Future directions:

Well-designed prospective, multicenter trials with standardized outcome measures should be the main focus of future research in order to enable insightful comparisons of anesthetic approaches. Clarifying safety profiles requires a stronger focus on long-term neurodevelopmental and maternal outcomes. It might be possible to lessen practice variability by creating precise clinical standards for choosing anesthesia in various emergency situations. The viability of rapid-onset neuraxial procedures for emergency cesarean deliveries may be improved by research into their optimization. Additionally, clinical judgment and the general treatment of obstetric anesthesia may be enhanced by the inclusion of patient-centered outcomes and the assessment of institutional training methods.
